# Oleic and Linoleic Acids Induce the Release of Neutrophil Extracellular Traps *via* Pannexin 1-Dependent ATP Release and P2X1 Receptor Activation

**DOI:** 10.3389/fvets.2020.00260

**Published:** 2020-06-05

**Authors:** Pablo Alarcón, Carolina Manosalva, John Quiroga, Isidora Belmar, Karina Álvarez, Gustavo Díaz, Anja Taubert, Carlos Hermosilla, María D. Carretta, Rafael A. Burgos, María A. Hidalgo

**Affiliations:** ^1^Laboratory of Inflammation Pharmacology, Faculty of Veterinary Sciences, Institute of Pharmacology and Morphophysiology, Universidad Austral de Chile, Valdivia, Chile; ^2^Laboratory of Immunometabolism, Faculty of Veterinary Sciences, Institute of Pharmacology and Morphophysiology, Universidad Austral de Chile, Valdivia, Chile; ^3^Faculty of Sciences, Institute of Pharmacy, Universidad Austral de Chile, Valdivia, Chile; ^4^Faculty of Veterinary Sciences, Universidad Austral de Chile, Valdivia, Chile; ^5^Institute of Parasitology, Biomedical Research Center Seltersberg, Justus Liebig University Giessen, Giessen, Germany

**Keywords:** PMN, purinergic receptor, ATP, non-esterified fatty acids, neutrophil extracellular trap

## Abstract

Non-esterified fatty acids (NEFAs) such as oleic acid (OA) and linoleic acid (LA) are associated with a higher incidence of infectious diseases such as metritis and mastitis during the bovine peripartum. Fatty acids can induce an increase in the release of ATP, and changes in the expression levels of purinergic receptors in bovine polymorphonuclears (PMN) during peripartum have also been reported. PMN respond to inflammatory processes with production of ROS, release of proteolytic and bactericidal proteins, and formation of neutrophil extracellular traps (NETs). NETs formation is known to require ATP production through glycolysis. Studies have shown that the above-mentioned metabolic changes alter innate immune responses, particularly in PMN. We hypothesized that NEFAs induce the formation of NETs through ATP release by Pannexin 1 and activation of purinergic receptors. In this study, we found that OA and LA induce NET formation and extracellular ATP release. Carbenoxolone, a pannexin-1 (PANX1) inhibitor, reduced OA- and LA-induced ATP release. We also found that *P2X1, P2X4, P2X5, P2X7*, and *PANX1* were expressed at the mRNA level in bovine PMN. Additionally, NEFA-induced NET formation was completely abolished with exposure to NF449, a P2X1 antagonist, and partially inhibited by treatment with etomoxir, an inhibitor of fatty acid oxidation (FAO). Our results suggest that OA and LA induce NET formation and ATP release *via* PANX1 and activation of P2X1. These new data contribute to explaining the effects of NEFA high concentrations during the transition period of dairy cattle and further understanding of pro-inflammatory effects and outcome of postpartum diseases.

## Introduction

In the transition period of dairy cows, which includes 3 weeks pre- and 3 weeks postpartum, there is a high demand for nutrients and a decrease in feed ingestion, producing a transient negative energy balance (1). This period is characterized by hypoglycemia, mobilization of non-esterified fatty acids (NEFAs), and an increase in plasma beta-hydroxybutyrate levels (2, 3). Several studies have shown that NEFA values in plasma increase progressively, peaking at 0.7 mM and in some cases reaching concentrations >1.5 mM during parturition (3–5). Moreover, around the time of parturition, high-yielding cows are predisposed to infectious diseases such as metritis (6) and mastitis (7), which is associated with an impaired innate immune response. Cows with high blood levels of NEFAs during peripartum have altered polymorphonuclear (PMN) trafficking and phagocytosis, as well as a reduced ability to kill microorganisms (8). This suggests that NEFAs can contribute to the modulation of the innate immune response, leading to the onset of metabolic and infectious diseases (8, 9).

Polymorphonuclear leukocytes cells (PMN) are the principal cell type of the host innate immune system and are regarded as the first line of defense against pathogens (10). PMN kill invading microorganisms primarily through three mechanisms: (1) phagocytosis and activation of NADPH oxidase with reactive oxygen species (ROS) production; (2) release of enzymes with proteolytic and bactericidal activities; and (3) release of fibers composed mainly of DNA, called neutrophil extracellular traps (NETs) (10, 11). Depending on the origin of the stimulus, NET production may or may not be dependent on NADPH oxidase activation (12). OA is a long-chain, monounsaturated fatty acid while LA is a long-chain, polyunsaturated fatty acid, and the levels of both NEFAs increase during the transition period in dairy cows (13). Both fatty acids are natural ligands for free fatty acid receptor 1 (FFAR1/GPR40), which is expressed in bovine PMN (14–16). Studies have shown that NEFAs activate bovine PMN responses *in vitro*. For instance, OA is reported to induce intracellular calcium mobilization, MAPK phosphorylation, superoxide production, and release of granules containing CD11b and MMP-9 (14, 16). Moreover, LA is known to increase MMP-9 release, stimulate PMN adhesion to endothelium, mobilize intracellular calcium, and activate signaling pathways such as ERK1/2 and p38 MAPK (17). However, the effects of OA and LA on NET production in cows are poorly understood.

In most mammalian cells, mitochondria are the main site for the aerobic oxidation of glucose and fatty acids, consumption of oxygen, and generation of ROS and ATP (18, 19). However, PMN have a few mitochondria and depend primarily on glycolysis for ATP production (20, 21). Besides being the primary source of cellular energy, ATP released into the extracellular space can also serve as important messenger molecules, facilitating communication between adjacent cells (21). Furthermore, extracellular ATP can serve as an autocrine signaling molecule through the activation of purinergic receptors (22). NET formation is known to require ATP production through glycolysis (23). Consistent with this, the mRNA expression of *P2X7, P2Y2*, and *P2Y11* was increased, whereas that of pannexin 1 (*PANX1*) was decreased, in bovine PMN 3 days postpartum (24), suggesting that ATP release and activation of purinergic receptors could be involved in bovine PMN activation in the peripartum period. Although fatty acids can induce an increase in the release of ATP (25), a few studies have investigated the effect of this nucleotide on bovine PMN activation.

Here, we examined whether purinergic signaling contributes to the increase in OA- and LA-induced NET release in bovine PMN. We observed that OA and LA increase ATP release through PANX1, contributing to the upregulation in NET production. In addition, our results support that beta-oxidation has an essential role in OA- and LA-induced NET extrusion, which suggests that fatty acid metabolism participates in the modulation of PMN responses.

## Materials and Methods

### Animals

Four Holstein Friesian heifers with body weights of 280–310 kg from the herd of the University Austral of Chile were used for the experiments. The heifers were fed twice a day with 1.0 kg of commercial concentrate, Cosetan® (IANSAGRO S.A., Chile), and grazed in naturalized pastures composed of *Holcus lanatus* and *Agrotis capillaris* pastures. Furthermore, a low contribution of forage legumes was administered, <10% of dry matter, and water *ad libitum*. All experiments were performed in strict accordance with protocols approved by the ethical committee of the Universidad Austral de Chile (permit number: 281/2017) and according to the current Chilean Animal Protection Laws.

### PMN Isolation

Blood was collected aseptically by jugular venipuncture into BD Vacutainer® tubes (Becton Dickinson, San Jose, CA, USA) containing acid citrate dextrose (ACD). The tubes were gently shaken for 5 min and then centrifuged at 1,000 × *g* for 20 min at 20°C. The plasma and buffy coat layers were aspirated and discarded, while the remaining red blood cells and PMN were resuspended in cold Hank's Balanced Salt Solution (HBSS; 5.0 mM KCl, 0.4 mM KH_2_PO_4_, 0.136 M NaCl, 0.3 mM Na_2_HPO_4_, and 0.6 mM D-glucose at pH 7.4). Blood was transferred to Falcon tubes (15 ml) and centrifuged again at 1,000 × *g* for 20 min at 20°C, and the remaining phlogistic layer was removed by aspiration with a Pasteur pipette. The erythrocytes were then separated twice by rapid hypotonic lysis with a cold, aqueous phosphate buffer solution (5.5 mM NaH_2_PO_4_ and 8.4 mM HK_2_PO_4_ at pH 7.2) in a 2:1 ratio. Next, isotonicity was restored with a solution of hypertonic phosphate (5.5 mM NaH_2_PO_4_, 8.4 mM HK_2_PO_4_, and 0.46 mM NaCl at pH 7.2) in a 1:1 ratio, and then centrifuged at 600 × *g* for 10 min at 20°C. The PMN pellet was resuspended and washed twice with cold HBSS, being centrifuged each time at 500 × *g* for 10 min at 20°C. Finally, the pellet was resuspended in 10 ml of cold HBSS and 100 μl of cells was incubated with 5 μM propidium iodide (Molecular Probes, Invitrogen, Carlsbad, CA, USA) in HBSS with Ca^2+^ for 5 min at room temperature (RT). After incubation, 900 μl of HBSS with Ca^2+^ was added, and purity, counts, and viability were assessed using flow cytometry (BD Accuri ™—BD, Franklin Lakes, NJ, USA; >94% purity and viability were used as thresholds for performing the experiments).

### Quantification of NETs by Fluorescence

PMN (1 × 10^6^) were suspended in HBSS with Ca^2+^ (0.897 mM) and exposed to 1 μM NF449 (P2X1 receptor antagonist; Tocris, Bristol, UK) (26), 10 μM carboxenolone (PANX1 inhibitor; Tocris) that was previously tested with different concentrations reported from former authors (27, 28), 0.1–50 μM 5-BDBD (P2X4 receptor antagonist; Tocris) (29), 0.001–10 μM A804598 (P2X7 receptor antagonist; Tocris) (30), 10 μM GW1100 (selective FFAR1 antagonist) (15, 16), and 10 μM DPI (NADPH oxidase inhibitor) (31) for 15 min at 37°C, except 5-BDBD that was incubated for 45 min, or 10 μM etomoxir [inhibitor of carnitine palmitoyltransferase-1 (CPT-1); Cayman Chemical, Ann Arbor, MI, USA] (32) for 60 min at 37°C. Then, OA (10–300 μM), LA (10–300 μM), or vehicle (0.01% DMSO) (15, 16), was added followed by incubation at 37°C for 30 min. Micrococcal nucleases (5 U/tube; New England Biolabs, Ipswich, MA, USA) were added and the PMN were incubated for 30 min. Then, the tubes were centrifuged at 800 × *g* for 6 min and 100 μl of the supernatant was transferred into transparent 96-well plates. Finally, 50 μl of PicoGreen (1:200, in HBSS with Ca^2+^; Invitrogen) was added. NETs were quantified using 485/520 nm excitation/emission wavelengths in a Thermo Scientific Varioskan Flash (Thermo Scientific, Waltham, MA, USA) and expressed as relative fluorescence units (RFUs). Measurements were performed in duplicate.

### Visualization of NET Release

PMN (2 × 10^5^) were preincubated with NF449 (1 μM) or carboxenolone (10 μM) for 15 min at 37°C, or with 10 μM etomoxir for 60 min at 37°C, and then stimulated with 300 μM OA or LA for 30 min. The PMN were fixed in a 2% paraformaldehyde solution for 30 min at RT, followed by blocking with a 1% BSA solution for 2 h, and incubation with an anti-histone H4 (citrulline 3) antibody (#07-596, Meck Millipore, Darmstadt, Germany) overnight at 4°C. The next day, samples were washed three times with sterile HBSS and incubated with an Alexa Fluor 405- or Alexa Fluor 488-conjugated anti-rabbit secondary antibody (#A31556 or A11055, Thermo Fisher Scientific, Waltham, MA, USA) for 2 h at RT. To visualize the nuclei, the covers were stained with PicoGreen (1:200 in HBSS with Ca^2+^) or Sytox Orange (5 μM in HBSS with Ca^2+^; #S34861, Thermo Fisher Scientific) for 30 min at RT. Images were acquired with a confocal microscope (Fluoview FV1000; Olympus, Tokyo, Japan).

### Quantification of ATP Levels

ATP levels were determined using a commercial ATP Determination Kit (#A22066, Thermo Fisher Scientific). PMN (5 × 10^5^) were resuspended in 250 μl of HBSS with Ca^2+^ and treated in two sets of experiments. In the first set, PMN were stimulated with 200 μM OA or LA, or vehicle (0.088% DMSO) for 0, 15, 30, or 60 s to obtain the time of the maximum ATP release for both NEFAs (**Figure 2**). In the second set, the PMN were treated with 10 μM carbenoxolone (CBX) or vehicle (0.01% DMSO) for 15 min at 37°C and then were stimulated with 300 μM OA or LA, or vehicle (0.088% DMSO) for 15 s (**Figures 3C,D**). Immediately after finishing the time of stimulation, the PMN were maintained on ice for 5 min. Subsequently, the cells were centrifuged at 600 × *g* for 5 min at 4°C. A 10-μl aliquot of the supernatant was incubated in 100 μl of the kit mix for the determination of ATP levels, according to the manufacturer's instructions. Finally, the samples were incubated at RT for 15 min, and the luminescence was measured using Varioskan Flash (Thermo Fisher Scientific). The data were normalized using the mean of 60 s of measurement of the control group.

### RT-qPCR

Total RNA was isolated from 5 × 10^6^ PMN per animal using EZNA Total RNA Kits (E.Z.N.A; Promega, Madison, WI, USA). Samples were treated with Turbo DNase-Free (Thermo Fisher Scientific). For cDNA synthesis, 500 ng of total RNA was reverse-transcribed using M-MLV Reverse Transcriptase Kits (Invitrogen, Thermo Fisher Scientific). Real-time PCR assays were performed using Takyon Rox SYBR® MasterMix dTTP Blue (Eurogentec, Fremont, CA, USA) and primers specific for bovine *P2X1*–*7, PANX1*, gap junction protein alpha 1 (*GJA1*), and housekeeping ribonucleoprotein S9 (*RPS9*). The primers used for the PCR reaction were as follows: P2X1 forward 5′-CTGTGCAGAGAACCCGGAAG−3′ and reverse 5′-CGTTGAAGGCCACACACTTG-3′; P2X2 forward 5′-GCGTTCTGGGACTACGAGAC-3′ and reverse 5′-CACGATGAACACGTACCACAC-3′; P2X3 forward 5′-CTACTTCGTGGGGTGGGTTT-3′ and reverse 5′-ATGACTCGGTTGGCATAGCG-3′; P2X4 forward 5′-GGTGCGTGTCATTCAATGGG-3′ and reverse 5′-AAAGCAGGCTTTGGCACTTC-3′; P2X5 forward 5′-GGGAGTGGGGTCTTTCTTCTG-3′ and reverse 5′-TTCACCTTTCCGTTCCCCTG-3′, P2X6 forward 5′-AACTTCAGGACAGCCACACA-3′ and reverse 5′-TGACCAGGATGTCGAAACGG-3′; P2X7 forward 5′-GCTGCAGCTGGAATGATGTTT-3′ and reverse 5′-AAAGAGCACCACGTGGAAGAG-3′; PANX1 forward 5′-TTGACTTGAGAGACGGTGCC-3′ and reverse 5′-TGGCTTTCCTGTGAACTTTGC-3′; GJA1 forward 5′-GAGTGCCTGGGCTTGCTTTT-3′ and reverse 5′-TTGCCTGGGTACTGCTCTTTCT-3′; and RSP9 forward 5′-GCTGACGCTGGATGAGAAAGACCC-3′ and reverse 5′-ATCCAGCACCCCGATACGGACG-3′. The following conditions were used on a StepOne Plus real-time PCR system (Thermo Fisher Scientific): one cycle at 95°C for 10 min and 35 cycles of 95°C for 15 s, 60°C for 30 s, and 72°C for 30 s. This was followed by analysis of dissociation (melting) curves to ensure primer specificity. Relative changes in gene expression were determined by the Δ^CT^ method (33). The efficiency of primers was obtained through StepOne software v2.3 (R^2^, Slope and percentage efficiency), and the efficiency was calculated as [10^(−1/slope)^]. To P2X1 R^2^ = 0.96; Slope = −3.30; % efficiency = 100%, and efficiency was 2.01. To P2X4 R^2^ = 0.99; Slope = −3.40; % efficiency = 95%, and efficiency was 1.97. To P2X5 R^2^ = 0.96; Slope = −3.50; % efficiency = 110%, and efficiency was 1.93. To P2X7 R^2^ = 0.99; Slope = −3.03; % efficiency = 110%, and efficiency was 2.14. Finally, to PANX1 R^2^ = 0.99; Slope = −3.30; % efficiency = 100%, and efficiency was 2.01.

### Statistical Analysis

The results are illustrated in bar graphs or dot plots as means ± S.E.M. of four independent experiments. One-way analysis of variance (ANOVA) was performed, and Fisher's LSD multiple comparison test was applied, using a significance level of 5%. When assumptions of normality or homogeneity of variance were not met according to the Shapiro–Wilks or Brown–Forsythe test, respectively, Kruskal–Wallis ANOVA and Dunn's multiple comparison test were used. All statistical analyses were performed using GraphPad Prism v7.0 (GraphPad Software, La Jolla, CA, USA). A *p*-value < 0.05 was considered significant.

## Results

### Oleic Acid and Linoleic Acid Induced NET Formation in Bovine PMN

**Figure 1:**
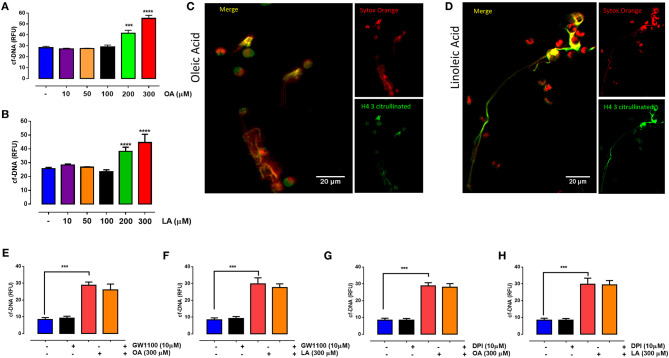
Oleic acid (OA) and linoleic acid (LA) induce neutrophil extracellular trap (NET) formation independently of FFAR1 and NADPH oxidase activation. Bar graph (means ± S.E.M.) showing the relative fluorescence (RFU) of cell-free (cf)-DNA obtained from PMN treated with different concentrations of OA **(A)** or LA **(B)**. Immunofluorescence of PMN treated with a 300 μM concentration of OA **(C)** or LA **(D)** using an anti-histone H4 citrulline 3 antibody as a NET marker and Sytox orange as a DNA marker. Images are representative of four independent experiments; scale bar = 20 μM. Bar graph of RFU (means ± S.E.M.) of cf-DNA obtained from PMN treated with GW1100 (FFAR1 antagonist) **(E,F)** or diphenyleneiodonium (DPI) **(G,H)** for 15 min and then stimulated for 30 min with OA or LA. *n* = 4; ***p < 0.001, ****p < 0.0001 compared with vehicle controls.

Exposure to 200 and 300 μM OA (Figures 1A,C) or LA (Figures 1B,D) increased NET formation after 30 min of stimulation. Moreover, the increase in NET formation induced by 300 μM OA and LA was completely abolished in the presence of DNase I (data not shown). To evaluate the mechanism by which OA and LA induce NET formation, PMN were exposed to GW1100, a selective FFA1 antagonist. The results showed that GW1100 treatment did not affect the OA- or LA-induced production of NETs (Figures 1E,F). Similarly, when PMN were incubated with DPI, a NADPH oxidase inhibitor, OA- or LA-induced NET formation was not affected (Figures 1G,H). These results suggested that the formation of NETs that was induced by short-term culturing with these fatty acids was independent of FFA1 and NADPH oxidase.

### An Increase in Extracellular ATP Levels Induced by Oleic Acid and Linoleic Acid Modulates NET Production

**Figure 2:**
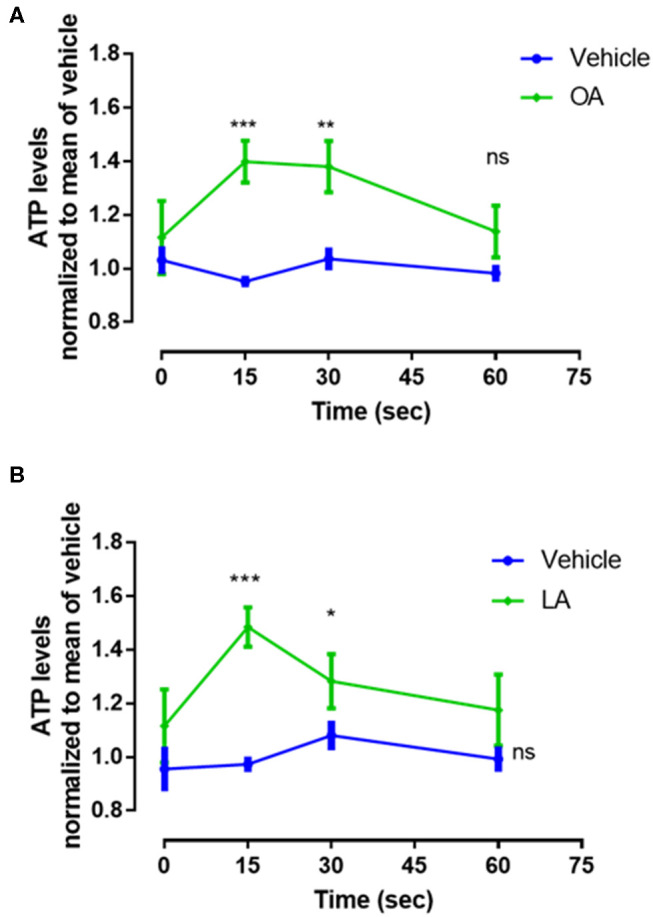
Oleic acid (OA) and linoleic acid (LA) induce ATP release. Levels of released ATP in supernatants of PMN treated with a 200 μM concentration of OA **(A)** or LA **(B)** for 0, 15, 30, and 60 s. The data were normalized using the mean of 60 s of measurement of control group. *n* = 4; *p < 0.05, **p < 0.01, ***p < 0.001 compared with vehicle controls.

To assess the mechanism associated with OA- or LA-induced NET formation, we measured the concentrations of extracellular ATP released within 60 s by PMN stimulated with 200 μM of both fatty acids. Our results showed that OA and LA induced a peak of ATP release between 15 and 30 s (Figure 2).

**Figure 3:**
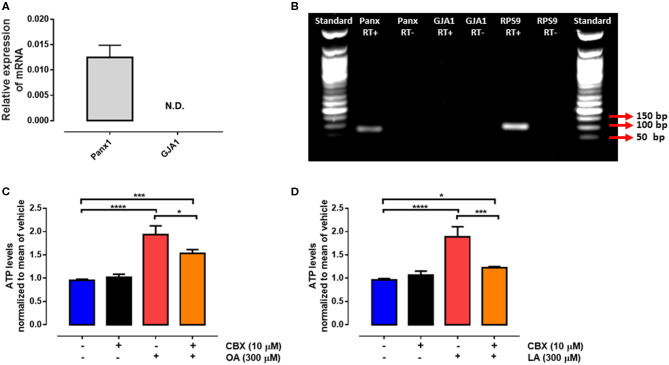
Non-esterified fatty acid (NEFA)-induced ATP release is mediated *via* PANX1. Relative expression of *PANX1* to RPS9 (housekeeping) in bovine PMN assessed by RT-qPCR **(A)**, *n* = 4. Gel of mRNA amplicons of *PANX1, GJA1*, and *RPS9* **(B)**. N.D.: not detected. bp: base pair. RT+, RT–: reverse transcriptase +, reverse transcriptase -. Bar graph showing ATP release from PMN treated with 10 μM carbenoxolone (CBX) for 15 min and then stimulated with oleic acid (OA) **(C)** or linoleic acid (LA) **(D)** for 30 min. *n* = 4; *p < 0.05, ***p < 0.001, ****p < 0.0001 compared with vehicle controls.

Pannexin-1 is an ATP release channel with important roles in both paracrine and autocrine ATP signaling (34). We found that *PANX1* mRNA was expressed in bovine PMN (Figures 3A,B). Furthermore, exposure to the PANX1 inhibitor CBX significantly decreased the levels of extracellular ATP induced by OA or LA (Figures 3C,D). These results suggest that both fatty acids induce an increase in extracellular ATP levels, and that this ATP is released through PANX1 in bovine PMN.

**Figure 4:**
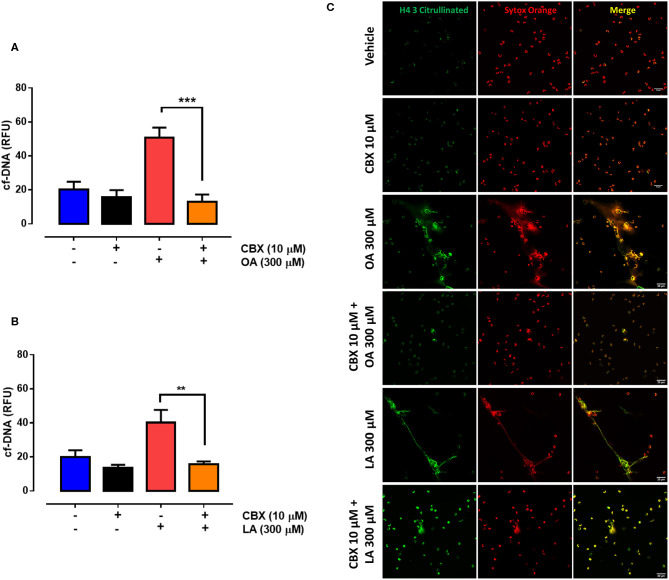
Oleic acid (OA) and linoleic acid (LA) induce neutrophil extracellular trap (NET) formation via PANX1-mediated ATP release. Bar graph (means ± S.E.M.) of cell-free (cf)-DNA of PMN treated with 10 μM carbenoxolone (CBX) for 15 min and stimulated or not with OA **(A)** or LA **(B)**. *n* = 4; **p < 0.01, ***p < 0.001 compared with OA or LA treatment alone. Representative image from four independent experiments of PMN treated with CBX and stimulated with OA or LA **(C)**.

ATP is an important extracellular ligand involved in autocrine signaling in PMN (35). To assess whether the PANX1-mediated increase in extracellular ATP levels is involved in fatty acid–induced NET formation, we evaluated the effect of CBX on NET production. We observed a significant decrease in OA- (Figure 4A) and LA-induced (Figure 4B) NET production in the presence of CBX. Similarly, cotreatment with CBX and either OA (Figure 4C) or LA (Figure 4D) led to a reduced number of structures such as NETs decorated with histone H4 citrulline 3 (H4Cit3) when compared with OA or LA treatment alone. All these results suggested that OA and LA induced ATP release and increased NET production through an autocrine signal.

### Expression of Purinergic P2X Receptors in Bovine PMN and Their Participation in NEFA-Induced NET Formation

**Figure 5:**
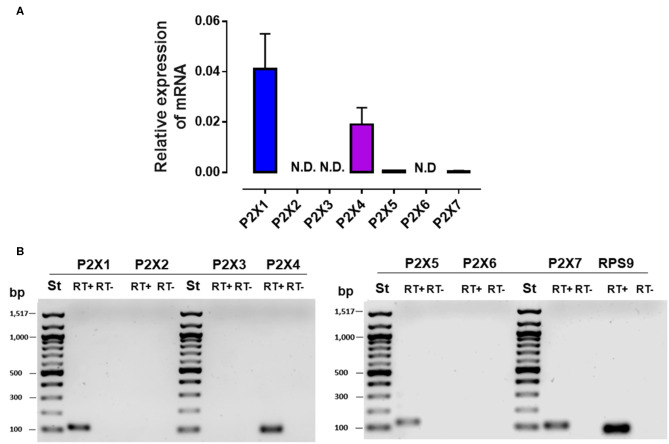
mRNA expression of the P2X receptor in bovine PMN. Relative expression of *P2X1*–*7* to RPS9 (housekeeping) in bovine PMN **(A)**. Representative image of PCR amplicons of P2X receptors **(B)**. N.D., not detected.; bp, base pair; ST, standard of bp; RT+, RT–, reverse transcriptase +, reverse transcriptase –. *N* = 4.

**Figure 6:**
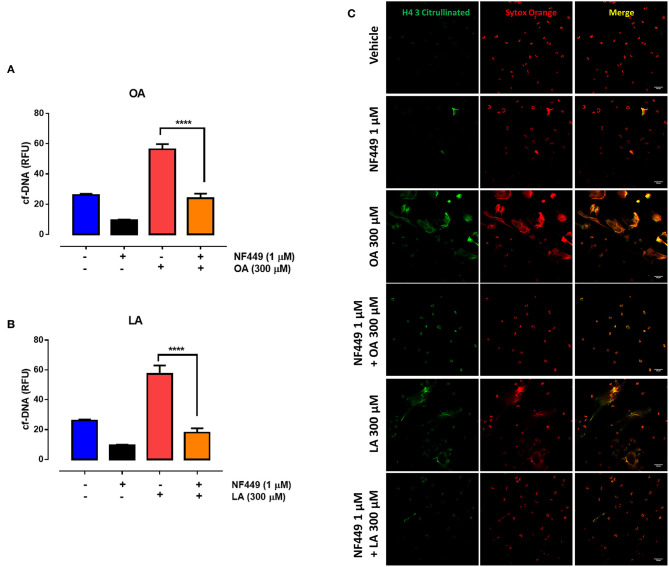
P2X1 is involved in oleic acid (OA)-induced neutrophil extracellular trap (NET) formation. Bar graph (means ± S.E.M.) of cf-DNA of PMN treated with 1 μM NF449 for 15 min and stimulated with OA **(A)** or linoleic acid (LA) **(B)**. ****p < 0.0001 compared with OA treatment alone. Representative images from four independent experiments of PMN treated with NF449 and stimulated with OA **(C)** or LA **(C)**. *n* = 4.

Recently, it was shown that extracellular ATP release by proinflammatory mediators can modulate PMN responses in an autocrine manner through the activation of the purinergic P2X receptor family members (21, 22). Therefore, we evaluated the expression of purinergic P2X receptors in bovine PMN. We found that *P2X1, P2X4, P2X5*, and *P2X7* were expressed in bovine PMN; however, the expression of *P2X1* and *P2X4* was higher than that of *P2X5* and *P2X7* (Figures 5A,B). To confirm the autocrine effect of ATP on the NEFA-induced production of NETs in bovine PMN, we evaluated the role of purinergic receptors in NET production using pharmacological inhibitors of P2X1, P2X4, and P2X7. We first determined the optimal inhibitory concentrations of the three antagonists and observed that treatment with NF449, a P2X1 inhibitor, significantly decreased the OA-induced release of cf-DNA at the concentrations of 1 and 0.5 μM (Supplementary Figure 1A). However, treatment with the P2X4 (Supplementary Figure 1B) and P2X7 (Supplementary Figure 1C) receptor inhibitors did not interfere cf-DNA release. Similarly, we did not observe any effect of the P2X4 and P2X7 antagonists with LA (data not shown). We did not test the effect of P2X5 on cf-DNA release as no specific antagonist for this receptor is yet available. Consistent with this finding, bovine PMN treated with 1 μM NF449 and exposed to OA or LA decreased cf-DNA release (Figures 6A,B) and NET formation (Figure 6C), compared with OA or LA treatment. These results suggest that exposure to NEFAs increases the production of NETs *via* the activation of purinergic signaling through P2X1 in bovine PMN.

### Beta-Oxidation Has a Role in OA-Triggered NET Induction

**Figure 7:**
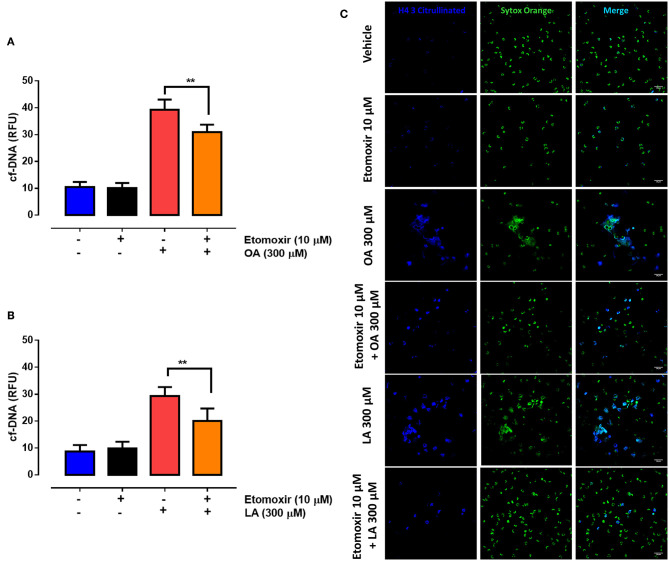
Etomoxir partially blocks non-esterified fatty acid (NEFA)-induced neutrophil extracellular trap (NET) formation. Bar graph (means ± S.E.M.) of cell-free (cf)-DNA of PMN treated with 10 μM etomoxir for 60 min and stimulated with oleic acid (OA) **(A)** or linoleic acid (LA) **(B)**. Representative image from four independent experiments of PMN treated with etomoxir and stimulated with OA or LA **(C)**. *n* = 4; **p < 0.01 compared with OA or LA treatment alone.

To determine whether OA and LA metabolism affects the production of NETs, we exposed PMN to etomoxir, an inhibitor of CPT-1, a key enzyme in the mitochondrial beta oxidation of fatty acids. We showed that etomoxir treatment partially decreased NET formation triggered by OA (Figure 7A) or LA (Figure 7B). In agreement with this, we observed a partial reduction in the number of NET-like structures decorated with H4Cit3 when the PMN were exposed to etomoxir and then treated with OA or LA (Figure 7C). Combined, the above results suggest that inhibition of the metabolic pathways of both fatty acids contributes to final NET release in OA- or LA-exposed PMN.

## Discussion

In this study, we evaluated whether NEFAs could induce NET release in bovine PMN and found that OA and LA can induce the formation of NETs staining positive for H4Cit3. This histone citrullination was previously described in NETs induced by D-lactate in bovine PMN (36), and is a key mechanism in NET production (37). A previous study indicated that OA at 17.7 μM (0.5 μg/0.1 ml) induces NET formation after 240 min of stimulus in human PMN (38). NET formation is also induced in mouse bone marrow–derived PMN stimulated with LA at 50 μM for 240 min (39). However, this response to OA and LA treatment was faster in bovine PMN (30 min). The formation of NETs in PMN was initially described as an effective antimicrobial mechanism that leads to the death of pathogens trapped within this extracellular DNA mesh (40). However, excessive NET production, especially surrounding healthy tissues, is harmful to the host and can lead to aseptic inflammatory processes (41, 42). Consequently, NETs were proposed to play both protective and pathogenic roles (43). In cattle and sheep, the presence of NETs has been observed in milk and the mammary gland (44, 45). On the other hand, the increase in NETs produces a cytotoxic effect on bovine mammary gland epithelial cells (BMECs) (46). These results have revealed that the increase in NETs contributes to the damage of BMECs associated with mastitis in bovine (46). In the uterus, there is a substantial influx of neutrophils after calving (around 40% PMN in cytology at day 7 postpartum) (47), in which one was exposed to high levels of NEFAs, thus with a reduced capacity of ROS production and NETs formation (48, 49), and the heifers are prone to acquire infectious diseases such as metritis and mastitis. Our results support the fact that NEFAs contribute to the increase in NETs, which is associated with a higher incidence of inflammatory diseases during the peripartum period in cattle.

Previous studies have indicated that the serum NEFAs concentrations reach values between 0.7 mM (5, 50) and 1.5 mM (4) approximately. Likewise, the percentage of OA and LA from serum total NEFAs fluctuates between 10 and 15% and between 26 and 40%, respectively, during bovine peripartum (5); thus, the OA and LA concentrations in peripartum with a high level of NEFAs (1.5 mM) could be between 150 and 225 μM and between 390 and 600 μM, respectively. The main limitation of NEFAs is linked to their very low aqueous solubility; hence, in *in vitro* studies, albumin can be added to partially bind to NEFAs to improve aqueous solubility. The fraction of unbound NEFAs accessible for cellular uptake depends on the ratio of total NEFAs to albumin (51). Therefore, the albumin concentration used may be critical in the biological effects of the FFA in *in vitro* studies. Furthermore, the affinity of the FFA for albumin will determine the concentrations of the unbound FFA and hence the effects observed (52). Despite the above, the molar ratio of FFA/albumin is often not reported, making it difficult to determine the proper concentration to obtain FFA-free levels for each *in vitro* model (51). Moreover, changes in albumin concentrations have been reported to influence the biological effects of the FFA (51). Nonetheless, in this work, the objective was to explain the mechanisms of the unbound FFA on the release of NETs; for this reason, we did not use albumin. However, it will be of great interest to evaluate these effects of the FFA in the presence of albumin in the near future to be as close as possible *in vivo*.

NET formation is known to be dependent on NADPH oxidase complex activation and ROS production (53). Moreover, OA and LA are reported to induce ROS production in bovine PMN through FFA1 activation (14, 15). However, we demonstrated that OA- and LA-induced NET formation was independent of FFA1 and NADPH oxidase activation, as treatment with GW1100 (FFA1 inhibitor) and DPI (NADPH oxidase inhibitor) did not reduce the levels of NEFA-mediated NET formation.

Previous studies have shown that treatment with OA and LA leads to increased ROS production in bovine PMN (14), while treatment with GW1100 results in decreased NEFA-induced ROS generation (15). Furthermore, NADPH oxidase inhibition by DPI also reduces NET formation induced by *Cryptosporidium parvum* (54), *Ostertagia ostertagi*, and PMA (55). *Leishmania* (56) and the calcium ionophore A23187 (57) have been reported to induce early NET formation *via* a ROS-independent mechanism, as has been the activation of SK3 potassium channels present on mitochondria (57).

Intense lipid mobilization from tissue stores is an essential metabolic adaptation during the transition period that results in a significant release of NEFAs into the bloodstream (2). Moreover, at 3 days postpartum in dairy cows, Seo et al. (24) demonstrated that PMN exhibit increased expression of PANX1 (24), a hemichannel involved in PMN-derived ATP release (22, 58). These observations suggest that, in this period, PMN can increase the extracellular ATP level *via* PANX1. We corroborated the presence of PANX1 in bovine PMN and further found that the NEFA-induced ATP release was inhibited by CBX, supporting a role for PANX1 in this innate immune response. Xiao et al. (25) demonstrated that palmitic acid induces ATP release via PANX1 in liver cells under hepatic inflammation and injury (25).

The ATP released into the extracellular space can serve as a messenger molecule that facilitates communications between adjacent cells (21). Moreover, an increase in ATP levels can act as a proinflammatory signal (59) and can also be classified as a “damage-associated molecular pattern” (DAMP) (60, 61). In connection with the above, extracellular ATP can function as an autocrine signaling molecule through the activation of purinergic receptors, as previously proposed (22).

Recently, PANX1-mediated ATP release from bone marrow–derived murine PMN was indicated to contribute to NET release independently of NADPH oxidase (62). Similarly, we demonstrated that CBX treatment reduced NEFA-induced NET formation, suggesting that ATP can act as an extracellular signal for NEFA-induced NET formation in bovine PMN.

ATP is a ligand for purinergic receptor family members in the innate immune system (60). The purinergic receptors P2X1, P2X4, P2X5, and P2X7 were shown to be expressed in both human (60, 63) and rat PMN (64). We found that these receptors were similarly expressed in bovine PMN, with P2X1 and P2X4 showing the highest expression levels. P2X receptors comprise a seven-member family (P2X1–7) of cation (Na^+^, K^+^, and Ca^2+^) channel receptors with differing functions in immune cells (65). In human PMN, P2X1 is involved in migration, degranulation, and phagocytosis (66). Other authors have clearly shown that P2X1 activation in neutrophils induces increased membrane expression of CD11b thereby promoting neutrophil migration from blood vessels into tissues (58, 67). Furthermore, it has been shown that activation of P2X1 by extracellular ATP can improve random migration of PMN through Rho kinase signaling (66). In addition to the above-mentioned, activation of P2X1 by ATP increases the entry of calcium in neutrophils (58). Transient calcium increase is necessary for neutrophil activation and exocytosis of cytosolic granules. Additionally, a recent study indicated that LPS-induced ATP release activates the release of secretory vesicles, tertiary granules, and secondary granules in autocrine form through P2X1 activation (66). Also, an efficient NETs formation required extra- and intracellular calcium mobilization (68). Finally, P2X1 activation has recently been involved in protozoan parasites *Besnoitia besnoiti* and *Neospora caninum*–induced NETosis in the bovine system (69, 70) proving its crucial role in PMN activation. P2X4 has been reported with a role in polarization, pseudopod formation, and migration of human T cells (71). However, it was not known whether P2X4 has a role in PMN function. Here, we showed that only P2X1 inhibition could reduce NET formation triggered by NEFAs.

As the NEFA-induced NET formation was independent of FFA1, other mechanisms may be involved, such as metabolic modulation. It well is known that PMN possess a highly developed mitochondrial network that is required for cellular processes such as chemotaxis and maintenance of cell shape and contribute to respiratory burst (72). Through FAO, NEFAs are broken down in peroxisomes or mitochondria to generate Acetyl-CoA, a metabolite that enters the tricarboxylic acid cycle or Krebs cycle to obtain ATP (73, 74), and this could be a source of ATP that could be released. Another source of ATP release is finely controlled by diffusion through plasmalemmal channels from exocytotic release from ATP-rich vesicles (75). Also, under extreme conditions, such as trauma, ischemia, and infection, cellular necrosis will release vast amounts of ATP from intracellular storage pools (76). Under basal conditions, mitochondria contribute mainly to cell death (apoptosis) and not contribute to energy metabolism in human PMN (20). However, Bao et al. (21) demonstrated that mitochondria regulate PMN activation by ATP production for autocrine signaling (21). In connection with the above, OA and LA can induce lipid droplet formation in beta-pancreatic cells (77) and human hepatic cells (Huh-7) (78). Besides, in a cell with a high metabolic rate like brown adipose tissue, it was found that mitochondria are associated to lipid droplet and increased pyruvate oxidation, electron transport, and ATP synthesis capacities (79), which one could be contributing to ATP release. It was recently proposed that fatty acids are metabolized by mitochondria through beta-oxidation and used as an energy source during PMN differentiation (80, 81).

We found that etomoxir, a beta-oxidation inhibitor, partially reduced NEFA-induced NET formation. This suggests that this metabolic pathway may contribute to NET release. Undifferentiated PMN show abundant lipid stores and increased FAO that may be critical for the supply of sufficient ATP for energy-demanding processes (80). Etomoxir was reported to partially block NET formation in immature rodent PMN (82), indicating that FAO contributes to the formation of NETs in PMN but may not be the only fuel source for this response.

In conclusion, we showed that NEFAs induce NET formation through PANX1-mediated ATP release and activation of P2X1 in bovine PMN. This phenomenon could explain the changes observed during the transition period of dairy cattle, characterized by elevated concentrations of NEFAs, disruption of immune and inflammatory functions, and appearance of postpartum diseases.

## Data Availability Statement

The datasets generated for this study are available on request to the corresponding author.

## Ethics Statement

The animal study was reviewed and approved by Universidad Austral de Chile (permit number: 281/2017).

## Author Contributions

PA, CM, RB, and MH designed the experiments. PA, CM, JQ, IB, KÁ, GD, AT, and CH performed the experiments. PA and CM prepared the manuscript. PA, CM, MH, and RB analyzed the data. All authors have read and approved the final version of this manuscript.

## Conflict of Interest

The authors declare that the research was conducted in the absence of any commercial or financial relationships that could be construed as a potential conflict of interest.

## Supplementary Material

Supplementary Figure 1 Bar graph (means ± S.E.M.) of cell-free (cf)-DNA of PMN treated with different concentrations of NF449 (potent and selective antagonist of the P2X1 receptor) **(A)** or 5-BDBD (P2X4 antagonist) **(B)** of A804598 (P2X7 antagonist) **(C)** and stimulated with OA. *N* = 4; ^**^*p* < 0.01, ^****^*p* < 0.0001 compared with OA treatment alone.

## References

[1] Wankhade PR Manimaran A Kumaresan A Jeyakumar S Ramesha KP Sejian V Metabolic and immunological changes in transition dairy cows: a review. Vet World. (2017) 10:1367–77. 10.14202/vetworld.2017.1367-1377 29263601 PMC5732345

[2] Contreras GA Sordillo LM Lipid mobilization and inflammatory responses during the transition period of dairy cows. Comp Immunol Microbiol Infect Dis. (2011) 34:281–9. 10.1016/j.cimid.2011.01.004 21316109

[3] McArt JA Nydam DV Oetzel GR Overton TR Ospina PA Elevated non-esterified fatty acids and beta-hydroxybutyrate and their association with transition dairy cow performance. Vet J. (2013) 198:560–70. 10.1016/j.tvjl.2013.08.011 24054909

[4] Veenhuizen JJ Drackley JK Richard MJ Sanderson TP Miller LD Young JW Metabolic changes in blood and liver during development and early treatment of experimental fatty liver and ketosis in cows. J Dairy Sci. (1991) 74:4238–53. 10.3168/jds.S0022-0302(91)78619-0 1787194

[5] Contreras GA O'Boyle NJ Herdt TH Sordillo LM Lipomobilization in periparturient dairy cows influences the composition of plasma nonesterified fatty acids and leukocyte phospholipid fatty acids. J Dairy Sci. (2010) 93:2508–16. 10.3168/jds.2009-2876 20494158

[6] LeBlanc SJ Interactions of metabolism, inflammation, and reproductive tract health in the postpartum period in dairy cattle. Reprod Domest Anim. (2012) 47 (Suppl. 5):18–30. 10.1111/j.1439-0531.2012.02109.x 22913557

[7] LeBlanc S Monitoring metabolic health of dairy cattle in the transition period. J Reprod Dev. (2010) 56 (Suppl):S29–35. 10.1262/jrd.1056S29 20629214

[8] Goff JP Major advances in our understanding of nutritional influences on bovine health. J Dairy Sci. (2006) 89:1292–301. 10.3168/jds.S0022-0302(06)72197-X 16537961

[9] Sordillo LM Contreras GA Aitken SL Metabolic factors affecting the inflammatory response of periparturient dairy cows. Anim Health Res Rev. (2009) 10:53–63. 10.1017/S1466252309990016 19558749

[10] Rosales C Neutrophil: a cell with many roles in inflammation or several cell types? Front Physiol. (2018) 9:113 10.3389/fphys.2018.00113 29515456 PMC5826082

[11] Mayadas TN Cullere X Lowell CA The multifaceted functions of neutrophils. Annu Rev Pathol. (2014) 9:181–218. 10.1146/annurev-pathol-020712-164023 24050624 PMC4277181

[12] Ravindran M Khan MA Palaniyar N Neutrophil extracellular trap formation: physiology, pathology, and pharmacology. Biomolecules. (2019) 9:365 10.3390/biom9080365 PMC6722781 31416173

[13] Adewuyi AA Gruys E van Eerdenburg FJ Non esterified fatty acids. (NEFA) in dairy cattle. A review. Vet Q. (2005) 27:117–26. 10.1080/01652176.2005.9695192 16238111

[14] Hidalgo MA Nahuelpan C Manosalva C Jara E Carretta MD Conejeros I Oleic acid induces intracellular calcium mobilization, MAPK phosphorylation, superoxide production and granule release in bovine neutrophils. Biochem Biophys Res Commun. (2011) 409:280–6. 10.1016/j.bbrc.2011.04.144 21575602

[15] Manosalva C Mena J Velasquez Z Colenso CK Brauchi S Burgos RA Cloning, identification and functional characterization of bovine free fatty acid receptor-1. (FFAR1/GPR40) in neutrophils. PLoS ONE. (2015) 10:e0119715 10.1371/journal.pone.0119715 25790461 PMC4366208

[16] Mena SJ Manosalva C Carretta MD Teuber S Olmo I Burgos RA Differential free fatty acid receptor-1. (FFAR1/GPR40) signalling is associated with gene expression or gelatinase granule release in bovine neutrophils. Innate Immun. (2016) 22:479–89. 10.1177/1753425916656765 27363707

[17] Mena J Manosalva C Ramirez R Chandia L Carroza D Loaiza A Linoleic acid increases adhesion, chemotaxis, granule release, intracellular calcium mobilisation, MAPK phosphorylation and gene expression in bovine neutrophils. Vet Immunol Immunopathol. (2013) 151:275–84. 10.1016/j.vetimm.2012.11.017 23267746

[18] Duchen MR Mitochondria in health and disease: perspectives on a new mitochondrial biology. Mol Aspects Med. (2004) 25:365–451. 10.1016/j.mam.2004.03.001 15302203

[19] Cruz MM Lopes AB Crisma AR de Sa RCC Kuwabara WMT Curi R Palmitoleic acid. (16:1n7) increases oxygen consumption, fatty acid oxidation and ATP content in white adipocytes. Lipids Health Dis. (2018) 17:55 10.1186/s12944-018-0710-z 29554895 PMC5859716

[20] Maianski NA Geissler J Srinivasula SM Alnemri ES Roos D Kuijpers TW Functional characterization of mitochondria in neutrophils: a role restricted to apoptosis. Cell Death Differ. (2004) 11:143–53. 10.1038/sj.cdd.4401320 14576767

[21] Bao Y Ledderose C Seier T Graf AF Brix B Chong E Mitochondria regulate neutrophil activation by generating ATP for autocrine purinergic signaling. J Biol Chem. (2014) 289:26794–803. 10.1074/jbc.M114.572495 25104353 PMC4175322

[22] Chen Y Yao Y Sumi Y Li A To UK Elkhal A Purinergic signaling: a fundamental mechanism in neutrophil activation. Sci Signal. (2010) 3:ra45 10.1126/scisignal.2000549 20530802 PMC4209711

[23] Yousefi S Stojkov D Germic N Simon D Wang X Benarafa C Untangling “NETosis” from NETs. Eur J Immunol. (2019) 49:221–7. 10.1002/eji.201747053 30629284

[24] Seo J Osorio JS Loor JJ Purinergic signaling gene network expression in bovine polymorphonuclear neutrophils during the peripartal period. J Dairy Sci. (2013) 96:7675–83. 10.3168/jds.2013-6952 24119811

[25] Xiao F Waldrop SL Khimji AK Kilic G Pannexin1 contributes to pathophysiological ATP release in lipoapoptosis induced by saturated free fatty acids in liver cells. Am J Physiol Cell Physiol. (2012) 303:C1034–44. 10.1152/ajpcell.00175.2012 22972801 PMC3492830

[26] Huang Z Liu P Zhu L Li N Hu H P2X1-initiated p38 signalling enhances thromboxane A2-induced platelet secretion and aggregation. Thromb Haemost. (2014) 112:142–50. 10.1160/TH13-09-0726 24633352

[27] Dembinska-Kiec A Pallapies D Simmet T Peskar BM Peskar BA Effect of carbenoxolone on the biological activity of nitric oxide: relation to gastroprotection. Br J Pharmacol. (1991) 104:811–6. 10.1111/j.1476-5381.1991.tb12511.x 1725764 PMC1908844

[28] Bruzzone R Barbe MT Jakob NJ Monyer H Pharmacological properties of homomeric and heteromeric pannexin hemichannels expressed in Xenopus oocytes. J Neurochem. (2005) 92:1033–43. 10.1111/j.1471-4159.2004.02947.x 15715654

[29] Layhadi JA Fountain SJ P2X4 receptor-dependent Ca(2+) influx in model human monocytes and macrophages. Int J Mol Sci. (2017) 18:2261 10.3390/ijms18112261 PMC5713231 29077063

[30] Janks L Sprague RS Egan TM ATP-Gated P2X7 receptors require chloride channels to promote inflammation in human macrophages. J Immunol. (2019) 202:883–98. 10.4049/jimmunol.1801101 30598517 PMC6352910

[31] Mejia SP Cano LE Lopez JA Hernandez O Gonzalez A Human neutrophils produce extracellular traps against *Paracoccidioides brasiliensis*. Microbiology. (2015) 161(Pt 5):1008–17. 10.1099/mic.0.000059 25701733

[32] Lambertucci RH Hirabara SM Silveira Ldos R Levada-Pires AC Curi R Pithon-Curi TC Palmitate increases superoxide production through mitochondrial electron transport chain and NADPH oxidase activity in skeletal muscle cells. J Cell Physiol. (2008) 216:796–804. 10.1002/jcp.21463 18446788

[33] Livak KJ Schmittgen TD Analysis of relative gene expression data using real-time quantitative PCR and the 2(-Delta Delta C(T)) Method. Methods. (2001) 25:402–8. 10.1006/meth.2001.1262 11846609

[34] Taruno A ATP release channels. Int J Mol Sci. (2018) 19:808 10.3390/ijms19030808 PMC5877669 29534490

[35] Bao Y Chen Y Ledderose C Li L Junger WG Pannexin 1 channels link chemoattractant receptor signaling to local excitation and global inhibition responses at the front and back of polarized neutrophils. J Biol Chem. (2013) 288:22650–7. 10.1074/jbc.M113.476283 23798685 PMC3829350

[36] Alarcon P Manosalva C Conejeros I Carretta MD Munoz-Caro T Silva LMR d(-) Lactic acid-induced adhesion of bovine neutrophils onto endothelial cells is dependent on neutrophils extracellular traps formation and CD11b expression. Front Immunol. (2017) 8:975 10.3389/fimmu.2017.00975 28861083 PMC5559443

[37] Neeli I Dwivedi N Khan S Radic M Regulation of extracellular chromatin release from neutrophils. J Innate Immun. (2009) 1:194–201. 10.1159/000206974 20375577 PMC6951038

[38] Khan MA Pace-Asciak C Al-Hassan JM Afzal M Liu YF Oommen S Furanoid F-acid F6 uniquely induces NETosis compared to C16 and C18 fatty acids in human neutrophils. Biomolecules. (2018) 8:144 10.3390/biom8040144 PMC6315434 30428625

[39] van der Windt DJ Sud V Zhang H Varley PR Goswami J Yazdani HO Neutrophil extracellular traps promote inflammation and development of hepatocellular carcinoma in nonalcoholic steatohepatitis. Hepatology. (2018) 68:1347–60. 10.1002/hep.29914 29631332 PMC6173613

[40] Papayannopoulos V Neutrophil extracellular traps in immunity and disease. Nat Rev Immunol. (2018) 18:134–47. 10.1038/nri.2017.105 28990587

[41] Gupta AK Hasler P Holzgreve W Gebhardt S Hahn S Induction of neutrophil extracellular DNA lattices by placental microparticles and IL-8 and their presence in preeclampsia. Hum Immunol. (2005) 66:1146–54. 10.1016/j.humimm.2005.11.003 16571415

[42] Kessenbrock K Krumbholz M Schonermarck U Back W Gross WL Werb Z Netting neutrophils in autoimmune small-vessel vasculitis. Nat Med. (2009) 15:623–5. 10.1038/nm.1959 19448636 PMC2760083

[43] Soderberg D Segelmark M Neutrophil extracellular traps in vasculitis, friend or foe? Curr Opin Rheumatol. (2018) 30:16–23. 10.1097/BOR.0000000000000450 28957962

[44] Reinhardt TA Sacco RE Nonnecke BJ Lippolis JD Bovine milk proteome: quantitative changes in normal milk exosomes, milk fat globule membranes and whey proteomes resulting from Staphylococcus aureus mastitis. J Proteomics. (2013) 82:141–54. 10.1016/j.jprot.2013.02.013 23459212

[45] Pisanu S Cubeddu T Pagnozzi D Rocca S Cacciotto C Alberti A Neutrophil extracellular traps in sheep mastitis. Vet Res. (2015) 46:59 10.1186/s13567-015-0196-x 26088507 PMC4471908

[46] Wei Z Wang J Wang Y Wang C Liu X Han Z Effects of neutrophil extracellular traps on bovine mammary epithelial cells *in vitro*. Front Immunol. (2019) 10:1003 10.3389/fimmu.2019.01003 31156617 PMC6533846

[47] Gilbert RO Santos NR Dynamics of postpartum endometrial cytology and bacteriology and their relationship to fertility in dairy cows. Theriogenology. (2016) 85:1367–74. 10.1016/j.theriogenology.2015.10.045 26944540

[48] Pascottini OB LeBlanc SJ Modulation of immune function in the bovine uterus peripartum. Theriogenology. (2020) 150:193–200. 10.1016/j.theriogenology.2020.01.042 31987594

[49] LeBlanc SJ Review: relationships between metabolism and neutrophil function in dairy cows in the peripartum period. Animal. (2020) 14:s44–54. 10.1017/S1751731119003227 32024567

[50] Drackley JK Veenhuizen JJ Richard MJ Young JW Metabolic changes in blood and liver of dairy cows during either feed restriction or administration of 1,3-butanediol. J Dairy Sci. (1991) 74:4254–64. 10.3168/jds.S0022-0302(91)78620-7 1787195

[51] Oliveira AF Cunha DA Ladriere L Igoillo-Esteve M Bugliani M Marchetti P *In vitro* use of free fatty acids bound to albumin: a comparison of protocols. Biotechniques. (2015) 58:228–33. 10.2144/000114285 25967901

[52] Spector AA Fatty acid binding to plasma albumin. J Lipid Res. (1975) 16:165–79. 236351

[53] Stoiber W Obermayer A Steinbacher P Krautgartner WD The role of reactive oxygen species. (ROS) in the formation of extracellular traps. (ETs) in humans. Biomolecules. (2015) 5:702–23. 10.3390/biom5020702 25946076 PMC4496692

[54] Munoz-Caro T Lendner M Daugschies A Hermosilla C Taubert A NADPH oxidase, MPO, NE, ERK1/2, p38 MAPK and Ca2+ influx are essential for *Cryptosporidium parvum*-induced NET formation. Dev Comp Immunol. (2015) 52:245–54. 10.1016/j.dci.2015.05.007 26026247

[55] Mendez J Sun D Tuo W Xiao Z Bovine neutrophils form extracellular traps in response to the gastrointestinal parasite *Ostertagia ostertagi*. Sci Rep. (2018) 8:17598 10.1038/s41598-018-36070-3 30514873 PMC6279769

[56] Rochael NC Guimaraes-Costa AB Nascimento MT DeSouza-Vieira TS Oliveira MP Garcia e Souza LF Classical ROS-dependent and early/rapid ROS-independent release of neutrophil extracellular traps triggered by Leishmania parasites. Sci Rep. (2015) 5:18302 10.1038/srep18302 26673780 PMC4682131

[57] Douda DN Khan MA Grasemann H Palaniyar N SK3 channel and mitochondrial ROS mediate NADPH oxidase-independent NETosis induced by calcium influx. Proc Natl Acad Sci USA. (2015) 112:2817–22. 10.1073/pnas.1414055112 25730848 PMC4352781

[58] Wang X Chen D Purinergic regulation of neutrophil function. Front Immunol. (2018) 9:399 10.3389/fimmu.2018.00399 29545806 PMC5837999

[59] Cauwels A Rogge E Vandendriessche B Shiva S Brouckaert P Extracellular ATP drives systemic inflammation, tissue damage and mortality. Cell Death Dis. (2014) 5:e1102 10.1038/cddis.2014.70 24603330 PMC3973196

[60] Junger WG Immune cell regulation by autocrine purinergic signalling. Nat Rev Immunol. (2011) 11:201–12. 10.1038/nri2938 21331080 PMC4209705

[61] Venereau E Ceriotti C Bianchi ME DAMPs from cell death to new life. Front Immunol. (2015) 6:422 10.3389/fimmu.2015.00422 26347745 PMC4539554

[62] Sofoluwe A Bacchetta M Badaoui M Kwak BR Chanson M ATP amplifies NADPH-dependent and -independent neutrophil extracellular trap formation. Sci Rep. (2019) 9:16556 10.1038/s41598-019-53058-9 31719610 PMC6851112

[63] Chen Y Corriden R Inoue Y Yip L Hashiguchi N Zinkernagel A ATP release guides neutrophil chemotaxis via P2Y2 and A3 receptors. Science. (2006) 314:1792–5. 10.1126/science.1132559 17170310

[64] Alkayed F Kashimata M Koyama N Hayashi T Tamura Y Azuma Y P2Y11 purinoceptor mediates the ATP-enhanced chemotactic response of rat neutrophils. J Pharmacol Sci. (2012) 120:288–95. 10.1254/jphs.12173FP 23182888

[65] Burnstock G Kennedy C P2X receptors in health and disease. Adv Pharmacol. (2011) 61:333–72. 10.1016/B978-0-12-385526-8.00011-4 21586364

[66] Wang X Qin W Xu X Xiong Y Zhang Y Zhang H Endotoxin-induced autocrine ATP signaling inhibits neutrophil chemotaxis through enhancing myosin light chain phosphorylation. Proc Natl Acad Sci USA. (2017) 114:4483–8. 10.1073/pnas.1616752114 28396412 PMC5410827

[67] Maitre B Magnenat S Heim V Ravanat C Evans RJ de la Salle H The P2X1 receptor is required for neutrophil extravasation during lipopolysaccharide-induced lethal endotoxemia in mice. J Immunol. (2015) 194:739–49. 10.4049/jimmunol.1401786 25480563

[68] Gupta AK Giaglis S Hasler P Hahn S Efficient neutrophil extracellular trap induction requires mobilization of both intracellular and extracellular calcium pools and is modulated by cyclosporine A. PLoS ONE. (2014) 9:e97088 10.1371/journal.pone.0097088 24819773 PMC4018253

[69] Villagra-Blanco R Silva LMR Munoz-Caro T Yang Z Li J Gartner U Bovine polymorphonuclear neutrophils cast neutrophil extracellular traps against the abortive parasite neospora caninum. Front Immunol. (2017) 8:606 10.3389/fimmu.2017.00606 28611772 PMC5447047

[70] Zhou E Conejeros I Velasquez ZD Munoz-Caro T Gartner U Hermosilla C Simultaneous and positively correlated NET formation and autophagy in besnoitia besnoiti tachyzoite-exposed bovine polymorphonuclear neutrophils. Front Immunol. (2019) 10:1131 10.3389/fimmu.2019.01131 31191523 PMC6540735

[71] Ledderose C Liu K Kondo Y Slubowski CJ Dertnig T Denicolo S Purinergic P2X4 receptors and mitochondrial ATP production regulate T cell migration. J Clin Invest. (2018) 128:3583–94. 10.1172/JCI120972 29894310 PMC6063471

[72] Fossati G Moulding DA Spiller DG Moots RJ White MR Edwards SW The mitochondrial network of human neutrophils: role in chemotaxis, phagocytosis, respiratory burst activation, and commitment to apoptosis. J Immunol. (2003) 170:1964–72. 10.4049/jimmunol.170.4.1964 12574365

[73] Eaton S Bartlett K Pourfarzam M Mammalian mitochondrial beta-oxidation. Biochem J. (1996) 320 (Pt 2):345–57. 10.1042/bj3200345 8973539 PMC1217938

[74] Han van der Kolk JH Gross JJ Gerber V Bruckmaier RM Disturbed bovine mitochondrial lipid metabolism: a review. Vet Q. (2017) 37:262–73. 10.1080/01652176.2017.1354561 28712316

[75] Eltzschig HK Sitkovsky MV Robson SC Purinergic signaling during inflammation. New Engl J Med. (2012) 367:2322–33. 10.1056/NEJMra1205750 23234515 PMC3675791

[76] Idzko M Ferrari D Eltzschig HK Nucleotide signalling during inflammation. Nature. (2014) 509:310–7. 10.1038/nature13085 24828189 PMC4222675

[77] Plotz T Hartmann M Lenzen S Elsner M The role of lipid droplet formation in the protection of unsaturated fatty acids against palmitic acid induced lipotoxicity to rat insulin-producing cells. Nutr Metab. (2016) 13:16 10.1186/s12986-016-0076-z PMC4766664 26918025

[78] Rohwedder A Zhang Q Rudge SA Wakelam MJ Lipid droplet formation in response to oleic acid in Huh-7 cells is mediated by the fatty acid receptor FFAR4. J Cell Sci. (2014) 127(Pt 14):3104–15. 10.1242/jcs.145854 24876224

[79] Benador IY Veliova M Mahdaviani K Petcherski A Wikstrom JD Assali EA Mitochondria bound to lipid droplets have unique bioenergetics, composition, and dynamics that support lipid droplet expansion. Cell Metab. (2018) 27:869–85.e866. 10.1016/j.cmet.2018.03.003 29617645 PMC5969538

[80] Riffelmacher T Clarke A Richter FC Stranks A Pandey S Danielli S Autophagy-dependent generation of free fatty acids is critical for normal neutrophil differentiation. Immunity. (2017) 47:466–80.e465. 10.1016/j.immuni.2017.08.005 28916263 PMC5610174

[81] Kumar S Dikshit M Metabolic insight of neutrophils in health and disease. Front Immunol. (2019) 10:2099 10.3389/fimmu.2019.02099 31616403 PMC6764236

[82] Hsu BE Tabaries S Johnson RM Andrzejewski S Senecal J Lehuede C Immature low-density neutrophils exhibit metabolic flexibility that facilitates breast cancer liver metastasis. Cell Rep. (2019) 27:3902–15.e3906. 10.1016/j.celrep.2019.05.091 31242422

