# Supplementary figures and images for: Oleic and Linoleic Acids Induce the Release of Neutrophil Extracellular Traps via Pannexin 1-Dependent ATP Release and P2X1 Receptor Activation
Source: Front Vet Sci. 2020 Jun 5;7:260. doi: 10.3389/fvets.2020.00260 (PMC7291836; doi:10.3389/fvets.2020.00260)

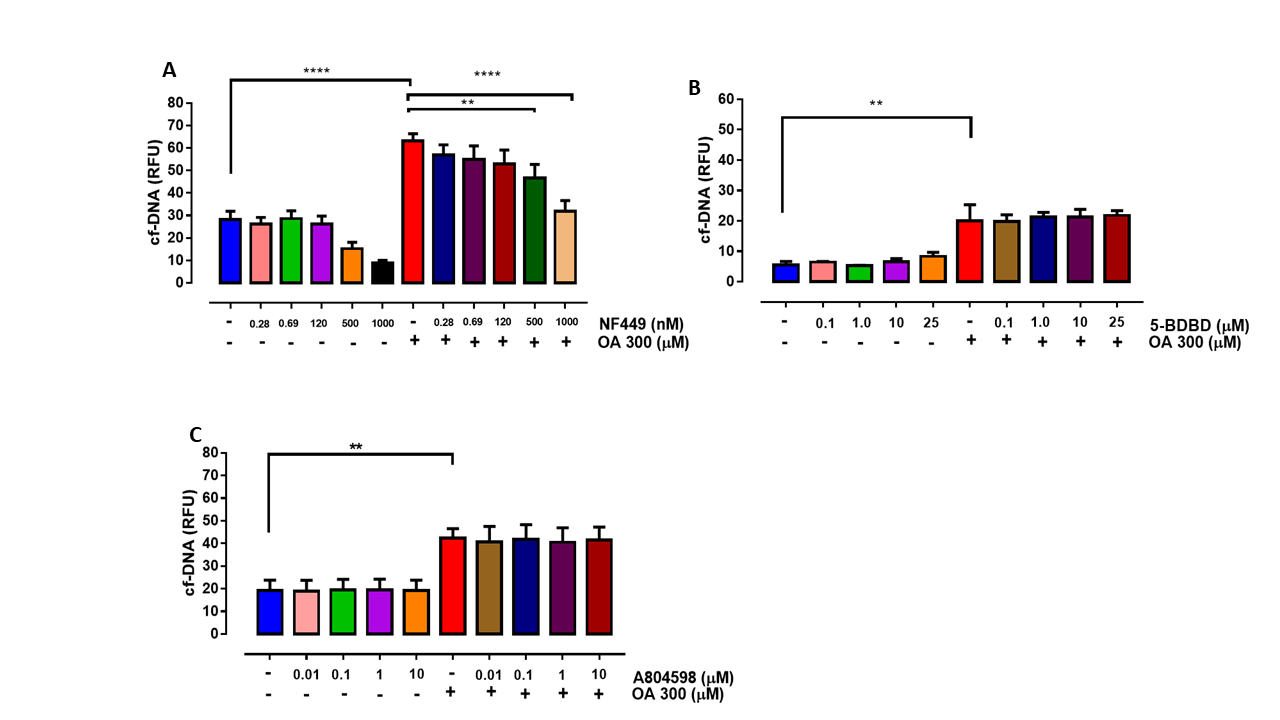

Supplement: Supplementary Figure 1 — Bar graph (means ± S.E.M.) of cell-free (cf)-DNA of PMN treated with different concentrations of NF449 (potent and selective antagonist of the P2X1 receptor) (A) or 5-BDBD (P2X4 antagonist) (B) of A804598 (P2X7 antagonist) (C) and stimulated with OA. N = 4; **p < 0.01, ****p < 0.0001 compared with OA treatment alone. [file Image_1.tif]
